# Varying intervals of antiretroviral medication dispensing to improve outcomes for HIV patients (The INTERVAL Study): study protocol for a randomized controlled trial

**DOI:** 10.1186/s13063-017-2177-z

**Published:** 2017-10-13

**Authors:** Risa Hoffman, Ashley Bardon, Sydney Rosen, Matthew Fox, Thoko Kalua, Thembi Xulu, Angela Taylor, Ian Sanne

**Affiliations:** 1 Division of Infectious Diseases, Department of Medicine, UCLA, 10833 Le Conte Ave, 37-121 CHS, Los Angeles, CA 90095 USA; 2 EQUIP, 3rd Floor Outspan Building, 1006 Lenchen North Avenue, Centurion, South Africa; 3 Department of Global Health, Boston University School of Public Health, 801 Massachusetts Ave, Crosstown Center, Boston, MA 02118 USA; 4 Health Economics and Epidemiology Research Office, Department of Internal Medicine, School of Clinical Medicine, Faculty of Health Sciences, University of the Witwatersrand, Johannesburg, South Africa; 5 Department of Epidemiology, Boston University School of Public Health, 801 Massachusetts Ave, Crosstown Center, Boston, MA 02118 USA; 6 Malawi Ministry of Health, P.O. Box 30377, Capital City, Lilongwe Malawi; 7 Right to Care, 3rd Floor Outspan Building, 1006 Lenchen North Avenue, Centurion, South Africa; 8 EQUIP-Zambia, 11059 Off Brentwood Road, Mikwala House, Longacres, Lusaka, Zambia; 9 Department of Medicine, Faculty of Health Sciences, University of the Witwatersrand, 1 York Ave, Parktown, 2193 South Africa

**Keywords:** Antiretroviral therapy, HIV, Africa, ART dispensing, Retention, Virologic suppression, Cost-effectiveness

## Abstract

**Background:**

Requirements for frequent dispensing of antiretroviral therapy (ART) place demands on health systems and can lead to suboptimal adherence and disengagement in care for patients due to the time and cost of frequent clinic visits. Rigorous data are needed to define optimal ART dispensing strategies and to evaluate the impact of a longer medication supply on retention and virologic suppression and determine whether this strategy lowers costs for both the patient and the health system. To date, no randomized studies have tested the benefits of 6-month dispensing of ART compared to 3-month and standard of care approaches.

**Methods:**

This study will be an unblinded cluster-randomized, matched controlled trial conducted among 8200 stable, HIV-infected individuals age 18 years and older on ART in Malawi and Zambia, to compare three ART dispensing intervals on the outcomes of retention in care (primary outcome), virologic suppression, and cost-effectiveness. Thirty clusters will be matched according to country, facility type, and ART cohort size and randomized to one of three study arms: standard of care, 3-month dispensing, and 6-month dispensing. Study participants will be followed, and outcomes will be measured at 12, 24, and 36 months. A subset of participants (n = 240) and providers (n = 180) will also participate in qualitative interviews to evaluate feasibility and acceptability of different ART dispensing intervals.

**Discussion:**

This study will be the first to compare 6-month and 3-month ART dispensing intervals for stable, HIV-infected individuals in Malawi and Zambia. We focus on outcomes relevant to country programs, including retention, virologic suppression, and cost-effectiveness. Results from the study will help resource-limited health systems better understand the full scope of outcomes resulting from various ART dispensing intervals and help to inform health policy decisions.

**Trial Registration:**

ClinicalTrials.gov, NCT03101592. Registered on 18 March 2017.

Pan African Clinical Trials, PACTR201706002336105. Registered on 2 June 2017.

**Electronic supplementary material:**

The online version of this article (doi:10.1186/s13063-017-2177-z) contains supplementary material, which is available to authorized users.

## Background

The 2015, the World Health Organization (WHO), the Joint United Nations Program on HIV/AIDS (UNAIDS), and the U.S. President’s Emergency Plan for AIDS Research (PEPFAR) all announced their support for global HIV control targets known as “90-90-90” [1, 2]. The goals of 90-90-90 are to identify 90% of people living with HIV, initiate 90% of HIV-diagnosed individuals on ART, and achieve viral suppression in 90% of those on ART. Modeling has indicated that achieving the 90-90-90 targets will require most low-income and middle-income countries to increase sharply the number of patients successfully retained on lifelong ART, with major implications for the resource requirements and costs of national treatment programs, and will heighten the challenge of maintaining program quality and improving patient outcomes as access expands.

Barriers to achieving the “third 90”—long-term viral suppression—include sub-optimal adherence and retention in care. For patients in sub-Saharan Africa, reasons for these poor outcomes have included the requirement for frequent clinic visits to receive medication refills, long wait times in ART clinics, high costs for travel to clinics, missed wages, and life events that lead to missed clinic visits [3–6]. Data from Zambia, a country with lower-middle income and high HIV prevalence in southern Africa, for example, suggest that requirements for monthly dispensing and/or guidelines that require multiple separate visits for refills and clinician evaluation impose a large burden on individuals living with HIV, which can in turn lead to treatment interruptions or complete disengagement from care [7].

One potential solution to this problem is to increase dispensing intervals to as long as 6 months to reduce patients’ visits to the clinic. An observational analysis of 130,000 patients in Zambia showed that patients on a 6-monthly appointment schedule were less likely than those on a monthly schedule to have gaps in medication refills (adjusted odds ratio (aOR) 0.50, 95% confidence interval (CI) 0.43–0.57) or be lost to follow up (aOR 0.48, 95% CI 0.40–0.59) [8]. Extension of refill intervals has been employed in many high-resource settings, including the USA, with supply provided up to every 6 months for stable patients [9, 10]. Potential benefits of multi-month dispensing may include improved adherence to ART, improved retention in care, and decongestion of clinics, which allows staff to focus on the sick, allows the clinic to initiate more new patients on ART, improves operational efficiency at clinics, and reduces costs of providing and obtaining ART.

Although the evidence from Zambia cited above is promising, it reflects the fact that longer dispensing intervals were almost certainly assigned to the patients with the best adherence records, and thus cannot tell us how multi-month dispensing affects outcomes. A review of the literature reveals no randomized studies of ART dispensing intervals in resource-limited settings and no cost estimates from real-world settings. Rigorous data are needed to define optimal dispensing strategies and to determine whether longer drug supply intervals result in improved outcomes and/or lower costs for both the patient and the health system.

The main objective of the INTERVAL study, a PEPFAR/US Agency for International Development (USAID)-supported, multi-site cluster-randomized trial, is to compare standard of care to 3-month and 6-month ART dispensing. The primary outcomes to be studied are retention in care at 12, 24, and 36 months. We will also evaluate secondary outcomes of virologic suppression and cost-effectiveness at these same time points. INTERVAL will contribute to the limited body of knowledge on multi-month dispensing of ART in resource-limited settings and may be used to better define best practices for cost-effective ART programs in Malawi, Zambia, and similar settings.

## Methods/design

INTERVAL will be a pragmatic, cluster-randomized, non-blinded, non-inferiority trial conducted at 30 health facilities in Malawi and Zambia. It will compare three ART dispensing strategies for stable patients – (1) standard of care, (2) 3-month ART dispensing, and (3) 6-month ART dispensing – on retention in care in Malawi and Zambia.

The study protocol was developed using the SPIRIT (Standard Protocol Items: Recommendations for Interventional Trials) Checklist (see Additional file 1) and adheres to the SPIRIT recommendations. The protocol has been approved by the institutional review board of the University of California, Los Angeles (UCLA), the National Health Sciences Research Council (NHSRC) in Malawi, the Zambia Excellence in Research Ethics and Science (ERES) Converge, the Zambia National Health Research Board (ZNHRB), and the Zambia Medicines Regulatory Authority (ZAMRA). The trial is registered with ClinicalTrials.gov as NCT03101592.

### Intervention

This study will compare standard of care to two alternative dispensing strategies. Under standard of care, ART dispensing intervals are based on provider opinion. Malawi and Zambia ART guidelines recommend 3-month dispensing for stable patients, but adherence to guidelines is variable and dispensing may vary due to provider and/or patient preference or medication availability. Participants in the 3-month dispensing study arm will receive a 90-day supply of ART and will have routine clinic visits every 90 days. Participants in the 6-month dispensing study arm will receive a 180-day supply of ART and will have routine clinic visits every 180 days. For all arms, other HIV services will be standard of care and supplies of co-trimoxazole (CPT) and isoniazid (IPT) will also be provided based on the assigned ART dispensing interval and country-specific guidelines about use of these therapies as part of routine HIV care.

Pharmacy support will be provided as part of the study, to ensure medication stock outs do not occur at study sites and that study sites do not take drug stocks away from non-study facilities. The trial is being conducted in close collaboration with the Ministries of Health in Malawi and Zambia and USAID, with support from the Supply Chain Management Systems (SCMS). Participants in the 6-month arm will be offered generic plastic bags to assist with carrying the large supply of medications. Participants will be given information about “ideal” basic storage conditions for medications, particularly in regard to temperature and avoidance of storage in direct sunlight. Participants who provide written informed consent will be willing to participate in any of the study arms; however, those in the 6-month arm may request a reduction in refill duration at any point during follow up. The proportion of individuals changing from 6-month ART dispensing to shorter intervals and reasons for changing will be captured during follow up.

To measure the primary outcome of retention, data will be abstracted from participants’ medical records. In Malawi and Zambia, default from care is defined as being out of ART for more than 60 days, and this definition will be used for the endpoint of retention (<60 days without ART at each time point).

**Fig. 1:**
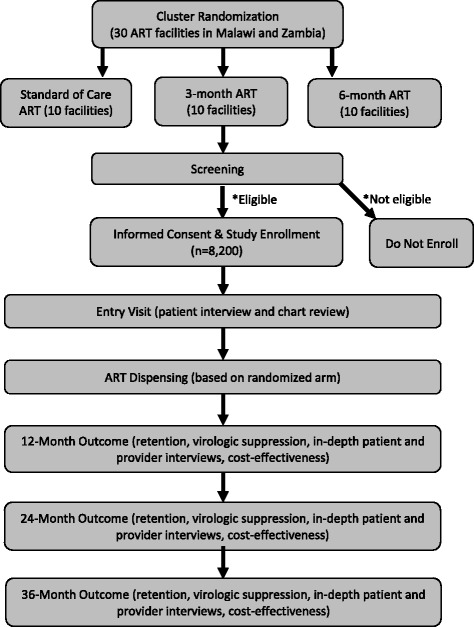
Flowchart of study protocol. *See Table 1. ART antiretroviral therapy

Secondary study outcomes include virologic suppression, cost-effectiveness, and feasibility and acceptability to patients and to providers and will be measured at 12, 24, and 36 months. Viral load data will be obtained from medical records based on any available data recorded during the prior year. Cost-effectiveness will be analyzed from both patient and health system perspectives by surveying participants at enrollment about time and costs associated with each clinic visit and by creating inventories of all resources and costs to the facility and health system for each patient visit. Feasibility and acceptability will be measured by conducting qualitative in-depth interviews with a subset of 240 participants (120 per country) and 180 providers (90 per country) evenly balanced among the three study arms. A flowchart of the study is provided in Fig. 1. Our main hypothesis is that 6-month ART dispensing will be non-inferior to 3-month intervals for retention and virologic suppression, but that 6-month intervals will be cost-effective compared to 3-month intervals.

### Study sites

The study will be conducted at 30 clusters in Malawi and Zambia. A cluster is defined as a hospital or health center that serves as an outpatient ART site. All clusters will either be government or mission facilities that provide free HIV care. Clusters will be selected based on ART cohort size and ability to enroll at least 271 participants, ability to perform viral load testing as part of routine care, completeness of medical records, and willingness to participate in the study. Sites in Malawi will be selected from the Central and Southern regions, and sites in Zambia will be selected from Central and Copperbelt Provinces. Sites participating in ongoing research that influences the frequency or location in which patients receive ART will be excluded, as will those that have active research underway on adherence and retention.

### Blinding and allocation

Random assignment of matched clusters will be determined by computer. Clusters will be matched based on available information that could potentially predict outcomes, including ART cohort size and region. Matched clusters will then be randomly allocated with one receiving the 3-month intervention, one the 6-month intervention, and one the control condition. Allocation will be completed by the study analyst. Clusters will then be informed of the allocation. No attempt will be made to blind the sites or patients to their allocated group, as both providers and patients will be aware of the amount of medication dispensed and the timing between appointments.

### Study population and sample size

HIV-infected individuals age 18 years or older who are considered stable on ART will be enrolled in the study. The definition of “stable on ART” and other inclusion and exclusion criteria are summarized in Table 1. Since there is no universally accepted definition of stability, criteria were developed for the study based on the expert opinion of infectious diseases specialists. As this is a cluster-randomized trial, study participants will receive the dispensing strategy that was randomly assigned to the cluster (health facility) at which they receive their HIV care.

**Table 1:** Study inclusion and exclusion criteria

Inclusion criteria
• Confirmed HIV-1 infection based on country standard of care for testing algorithm
• Stable on ART, defined as:
− on ART for at least 6 months
− on a first-line ART regimen as defined by country-specific guidelines (efavirenz, tenofovir, and emtricitabine or lamivudine)
− no drug toxicity/tolerability issues within the prior 6 months
− no period of more than 1 month without medication possession within the last 6 months
− no active opportunistic infection (OI) suspected (including tuberculosis (TB)) and not treated for an OI within the last 30 days
− undetectable viral load (as defined by country guidelines) within the last 6 months:
▪ Malawi: less than 1000 copies/mL
▪ Zambia: less than 20 copies/mL
• Willing and able to provide written informed consent
• Planning to receive HIV care from the same facility for at least 1 year
Exclusion criteria
• Co-morbid condition(s) for which the individual is treated at the ART clinic (hypertension, diabetes mellitus, chronic lung disease, etc.)
• Pregnant or breastfeeding, or if not breastfeeding, less than 6 months postpartum
• Already receiving care within a differentiated model in which care is received as a group or in a community, such as a community-based adherence group or adherence club
• Enrolled in any other research studies that would influence ART adherence, retention, or dispensing interval

### Sample size

Our study sample size was estimated for a cluster-randomized non-inferiority trial. With 30 clusters available for randomization, we estimated the sample size assuming a fixed number of clusters (*k*), an equal number of clusters per arm, and an equal number of subjects per cluster. Using a one-sided *Z* test (unpooled) with a significance level of 0.05, we estimated that about 5% of subjects would fail to be retained in care in the standard of care arm and 7.5% to be non-inferior in the 3-month or 6-month study arms. Assuming power of 90% and an intracluster correlation coefficient (ICC) of 0.004 [11], we will need to enroll 271 subjects per cluster for a total of 2710 subjects per arm and 8130 total individuals.

### Study procedures and data collection

**Fig. 2:**
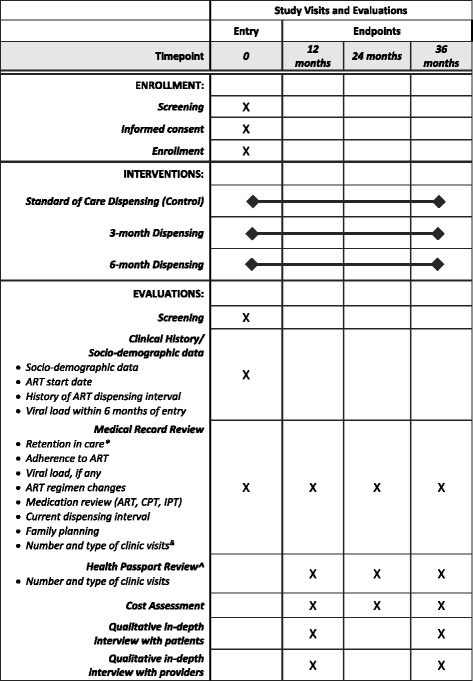
SPIRIT Figure. *Default defined as out of antiretroviral therapy (ART) for more than 60 days; ^&^Zambia only; ^^^Malawi only; CPT: co-trimoxazole prophylaxis therapy; IPT: isoniazid preventive therapy

The study procedures and data collection methods are described in detail below. A summary of the study visits and evaluations is outlined in the SPIRIT Figure (Fig. 2) below.

#### Eligibility screening

Patients will be provided general information about the study in ART clinic waiting areas and recruited by a study staff representative. Interested patients will be taken to a private space with a study staff representative to learn about the study, provide oral consent, undergo anonymous screening procedures, and, if eligible, provide written informed consent. A recent viral load test (within 6 months of the entry visit date) is required to determine eligibility; however, we anticipate many participants will not have a recent viral load result available at the time of screening. For participants who do not have a viral load documented, a sample will be collected after written informed consent is obtained, but the participant will not be enrolled in the study until the test results have been received. Patients who have a viral load sample drawn for study eligibility will receive test results during their next routine visit and will repeat the screening survey at that visit to ensure there have been no changes in eligibility criteria over the interim period.

#### Entry visit data collection

All patient baseline data will be collected in the entry visit, which may come immediately after screening for eligible patients, or may take place at the next visit when a viral load result is available. Two types of data will be collected at the entry visit. First, the participant’s medical record will be reviewed to collect clinical history, including date of HIV diagnosis, date of ART initiation, use of CPT and/or IPT, and history of ART clinic visits and ART dispensing intervals in the prior 12 months. The second type of data will come from an interview with the patient. In the interview, the study interviewer will obtain sociodemographic information about the participant, including gender, highest level of education, household composition, employment status, HIV disclosure status, and travel and opportunity costs related to the participant’s ART clinic visits. After completion of the entry visit data collection, the participant will complete her or his routine clinic visit with site providers and receive ART dispensing as per the site randomization. If CPT and/or IPT dispensing is standard of care for the facility, CPT and/or IPT will also be provided as per the randomized dispensing interval.

#### Follow-up data collection

Retention and virologic suppression will be assessed 12, 24, and 36 months after the entry visit date. At each of these time points, data from participants’ medical records will be collected by study staff. Data will include number, timing, and reason for clinic visits, retention in care (and if not retained, reason), possession of ART based on number of pills dispensed at each visit, possession of CPT and/or IPT based on number of pills dispensed at each visit, and viral load. Study staff will allow up to 60 days after a time point has been reached before medical record review, allowing for participants to meet the protocol-defined definition of default. For participants who default, facility standard of care procedures for tracing may be used, but the study will not make contact with the participant for the purposes of facilitating return to care.

#### Qualitative data collection

To help understand patients’ and providers’ perceived feasibility and acceptability of the dispensing intervals offered in the study, qualitative, semi-structured, in-depth interviews will be conducted at the study sites for both patients and providers after the 12-month primary endpoint has been reached. For study participants, a subset (up to 40 from each arm in Malawi and another 40 from each arm in Zambia, or a total of up to 240 individuals) will be randomly selected to participate in an individual, in-depth, qualitative interview. Participants will only be contacted for an interview if they provide informed consent for future contact at the time of initial written informed consent. Those who agree to an interview will return to the clinic for this step and will be reimbursed transport costs for this study visit. The interview will be conducted in a private room at the clinic in a local language and will last approximately 1 hour. Participants will be asked about their experiences receiving their assigned dispensing interval, including challenges in transporting medications from clinic, challenges in storing medications at home, pressure to sell or give ART supply to others, clinic usage outside of ART refills, and perceived financial challenges that result from clinic visits.

There is a possibility that the interviews may influence participants’ future outcomes in the study, particularly retention in care. Patients may change their behavior as a result of more intensive contact with study staff, because of being reimbursed for a study visit, or as a result of information discussed in the interview. The total number of individuals participating in interviews is less than 3% of the total study population, and these individuals will be excluded from outcome analyses after the first year.

We will also collect qualitative data from a subset of providers (n = 30) drawn from lists provided by the study sites from each of the three study arms in each country (total n = 180). These interviews will be performed after 50% of the facility’s study participants have reached the 12-month endpoint. For providers who agree to participate, study staff will complete an anonymous screening survey to determine eligibility to participate in the interview. If eligible, providers will be asked for written informed consent. The interviews will be conducted in English in a private space at the facility. Questions will focus on provider perceptions of challenges faced by patients in transporting or storing medications; perceptions of the degree to which patients lose, sell, or give away ART; issues related to medication stock and storage within their facility; and perceptions about the effect of ART dispensing interval on clinic efficiency and health system costs.

### Cost-effectiveness data collection

To estimate the provider costs of the different dispensing intervals per patient treated, we will create an inventory of all resources used to achieve the observed study outcomes from study enrollment to the 12-month, 24-month, and 36-month time points, including ART medications, non-ART medications, laboratory tests, outpatient clinic visits, other clinic services provided, inpatient care visits, fixed costs of patient care, and depreciated investment costs of establishing capacity for multi-month dispensing (e.g., medication storage capacity, pharmacy infrastructure, staff training, etc.). Data will be abstracted from patients’ medical records and the site’s performance reports (e.g. for patient volumes served) and finance and procurement records (for unit costs). Where necessary, cost data will also be obtained from national sources (e.g. Ministry of Health salary scales), commercial price lists, other cost-related studies underway in the INTERVAL countries, and published sources.

Fixed or indirect costs at the facility level include space, utilities, shared staff such as data clerks or managers, and other resources that do not vary directly with patient numbers, and will be allocated to study patients based on the proportion of total facility resources utilized for ART clinic visits. For example, if 10% of a clinic’s total number of visits per month is for ART patients, then 10% of overall clinic fixed costs will be allocated to ART and divided by the total number of ART patients to estimate a fixed cost for each clinic. If data on monthly clinic visits are not available, fixed costs will be allocated on the basis of space utilization. Data on costs incurred by patients for each of the arms will be collected through the entry visit survey, as described above. We will not estimate costs incurred above the level of individual facilities, such as those incurred by the government for overall HIV program management.

### Data safety and monitoring plan

The first data analysis will be performed when 50% of participants have reached 12 months of follow up. The study will be stopped early if we find a difference (at 12 or 24 months) between 3-month versus 6-month dispensing with a *p* value <0.01. Operational futility may be considered if the observed accrual patterns are exceedingly different than planned, and the protocol team has had a chance to address the shortcomings of accrual.

### Data analysis

#### Primary outcome

Our primary outcome, which is the basis for our sample size estimates and primary analysis, is retention in care at 12 months after study enrollment. Data analyses will begin with descriptive measures about the study population stratified by treatment arm and by facility and presented as medians and interquartile ranges for continuous measures and proportions for categorical variables. These measures will be used to look for large variations by facility and for imbalances between study arms.

We will perform an intention-to-treat analysis for the primary outcome of retention. Our analysis will use a log-linear generalized estimating equation to estimate the risk ratios and associated 95% confidence interval for the effect of each intervention arm compared to standard of care. We will specify facility-level clustering to account for the study design and estimate robust standard errors using an unstructured correlation matrix. Should we identify any baseline imbalances between study arms, we will adjust for these in our multivariable model and report adjusted risk ratios and corresponding 95% confidence intervals.

#### Secondary outcomes

Our study has not been powered to look at secondary aims, but we expect to have enough power to explore differences in secondary endpoints. The analytic methods for the secondary outcome of virologic suppression will be identical to those described above for the primary outcome of retention. For qualitative secondary outcomes of patient and provider feasibility and acceptability of dispensing intervals, interviews will be transcribed by trained personnel. Interviews will be analyzed in Atlas.ti v.6.2 using thematic analysis. Two investigators will code the first five to ten interviews by themes and compare coding to reach a consensus. A codebook will be developed, and two individuals will independently code all transcripts and compare codes to reach a consensus. For each theme, we will describe the range, central tendency, and context in which each theme emerges. This process will be done separately for participants and providers.

Cost-effectiveness will be assessed as the average cost per patient achieving the primary outcome in each arm. Cost will be estimated using micro-costing methods developed by the investigators and widely published [12–14]. Costs will be reported as means with 95% confidence intervals and medians with IQRs. Using the average cost per patient, we will then estimate the cost per outcome achieved in each arm. We will compare average cost/patient retained in care across the three study arms, initially at 12 months and again at 24 and 36 months. The annual cost of providing ART under each of the three strategies evaluated (independent of outcomes) for the overall HIV program budget in each country will be estimated by combining the cost per patient treated under each strategy, study data on the proportions of all ART patients at study clinics who are eligible for each dispensing interval, and up-to-date, published or reported estimates of total numbers of patients on ART in each country.

To estimate the costs to patients of obtaining ART, we will use baseline patient interview data to calculate an average cost per clinic visit, including transport fares, food and accommodation while away from home, lost wages, and substitute labor. For each participant, the number of clinic visits made per 12-month period for any HIV-related reason will then be multiplied by the cost per visit. We will estimate and compare the average cost per patient of obtaining care in each study arm.

Finally, we will explore clinic resource allocation to determine the effects of the dispensing interval on patients’ access to care. We will use two proxy measures to analyze access. First, we will estimate and compare the average waiting time and/or total time in clinic among patients seeking HIV care in each arm of the study. Second, we will use aggregate, clinic-level data to ascertain whether the quantity or mix of services provided by each study site changes between the last month before the start of the study and the first month of year 2 of the study. While neither of these measures is a perfect indicator of access, both will provide some indication of the availability of both HIV care and care for non-HIV conditions.

## Discussion

To date, no randomized studies have been conducted to evaluate the benefits and challenges associated with longer ART dispensing intervals in resource-limited settings. This study will be the first to compare standard of care, 3-month, and 6-month ART dispensing intervals for stable, HIV-infected individuals in Malawi and Zambia. We focus on outcomes that are relevant to national HIV programs and that could result in widespread policy changes. Results from the study will help limited-resource health systems better understand the full scope of outcomes resulting from various ART dispensing intervals and help to inform health policy decisions. Our study is innovative in that it includes qualitative data collection from patients and providers. These data can help to address questions about moving to longer dispensing intervals, including around patient experiences transporting a large supply of medications (6 months of ART, CPT, and/or IPT), patient challenges with storage of these large supplies, particularly for those who have not disclosed to other members living in the same household, and concerns that patients given a large supply of ART may be inclined to sell medications for income or provide them to friends or family. We will also estimate both total costs of implementation and cost-effectiveness. Cost savings are widely anticipated from adopting longer follow-up intervals, but such savings have yet to be demonstrated or quantified in real-world settings, making the economic evaluation essential for resource allocation decisions.

This study has several limitations. Apart from the entry viral load, the study will not perform follow-up viral load testing. Therefore, results for the secondary outcome of viral load will be limited to those tests performed within the context of routine care. In Zambia, viral load monitoring is performed annually, and in Malawi, monitoring occurs every 2 years. We anticipate that viral load results may be missing from a large number of participants as these programs are both early in their viral load scale-up efforts. Because of these concerns, viral suppression is a secondary outcome. Our definition of a “stable” ART patient was developed in conjunction with HIV experts and takes into consideration published literature on this topic [2, 15–17]. Our results may not be generalizable in settings that use a different definition of stable. There may be a subset of unstable ART patients who struggle with retention because of circumstances that prevent frequent follow-up to an ART facility (living remotely, difficult work schedule, school or work outside of the country). These individuals could benefit from a 6-month dispensing interval, but they will not be included in our study, and their outcomes will not be captured.

Participant blinding will not be used in the trial as there is no way to blind providers or patients to their assigned group. Lack of blinding could potentially influence the study results as participants who are motivated to have a longer drug supply could transfer to a study facility with 6-month dispensing. Additionally, regardless of arm, participants may be more likely to be retained if they know they are being followed as part of a study. We have attempted to reduce bias by limiting interaction with study participants to the screening and entry visits, which will both coincide with routine ART visits. All subsequent data will be obtained from medical record review with the exception of a small subset of participants (n = 240 across both countries) who will be asked to complete in-depth interviews at the end of the first year. These limitations will be noted in study findings when they are presented.

It is possible that Malawi and Zambia will revise their HIV treatment guidelines during study follow up, including extending dispensing intervals to 4 months or longer. In Malawi, IPT rollout in high-burden TB districts is anticipated during the first year of the study, and this will result in patients returning to clinic for extra visits to adhere to the IPT monitoring schedule (monthly for the first 3 months of treatment). These national guideline changes will be captured at the level of individual participant data and accounted for in our analyses, but they may influence our ability to measure intended study outcomes. Finally, the study will be working alongside Ministry of Health in both Malawi and Zambia to ensure that sites randomized to 6 months will have adequate supplies of ART, CPT, and IPT. Should countries adopt 6-month dispensing, this high level of support would be unlikely to be available at all sites, resulting in potential challenges with procurement, supply chain, and/or storage.

Despite these limitations, this study will be the first randomized effort to measure multi-month ART dispensing intervals on the outcomes of retention, virologic suppression, and cost-effectiveness. If 6-month dispensing intervals are proven to be non-inferior and cost-effective, the results may help to inform ART dispensing standard of care guidelines in Malawi, Zambia, and other limited-resource health systems.

## Trial status

This manuscript was developed using study protocol version 2.4, 22 June 2017, for “The INTERVAL Study: Varying Intervals of ART Dispensing to Improve Outcomes in HIV”. Recruitment and enrollment began 31 May 2017. Enrollment is expected to be completed by 30 November 2017.

## Additional file

Additional file 1: SPIRIT 2013 Checklist: Recommended items to address in a clinical trial protocol and related documents*. (DOCX 52 kb)

## Abbreviations

aOR: Adjusted odds ratio
ART: Antiretroviral therapy
CI: Confidence interval
CPT: Co-trimoxazole prophylaxis therapy
HIV: Human immunodeficiency virus
ICC: Intracluster correlation coefficient
IPT: Isoniazid preventive therapy
IQR: Interquartile range
OI: Opportunistic infection
PEPFAR: U.S. President’s Plan for Emergency AIDS Relief
TB: Tuberculosis
USAID: United States Agency for International Development
